# Resveratrol as a Therapeutic Agent in Alzheimer’s Disease: Evidence from Clinical Studies

**DOI:** 10.3390/nu17152557

**Published:** 2025-08-05

**Authors:** Nidhi Puranik, Meenakshi Kumari, Shraddha Tiwari, Thakur Dhakal, Minseok Song

**Affiliations:** 1 Department of Life Sciences, Yeungnam University, Gyeongsan 38541, Republic of Korea; nidhipuranik30@gmail.com (N.P.); shraddha_tiwari@yu.ac.kr (S.T.); thakurdhakal2003@gmail.com (T.D.); 2 Department of Botany, Career Point University, Kota 324005, Rajasthan, India

**Keywords:** AD, polyphenolic compound, resveratrol, drug delivery systems, Tau protein, amyloid-beta (Aβ) plaques

## Abstract

Alzheimer’s disease (AD) is a progressive neurodegenerative disorder characterized by cognitive decline, memory loss, and neuronal dysfunction. It is driven by the accumulation of amyloid-beta (Aβ) plaques, Tau protein hyperphosphorylation, oxidative stress, and neuroinflammation. Resveratrol (RSV) is a natural polyphenolic compound found in grapes, berries, and red wine that has garnered attention for its potential neuroprotective properties in combating AD. The neuroprotective effects of RSV are mediated through the activation of sirtuins (SIRT1), inhibition of Aβ aggregation, modulation of Tau protein phosphorylation, and the attenuation of oxidative stress and inflammatory responses. RSV also enhances mitochondrial function and promotes autophagy, which are important processes for maintaining neuronal health. Preclinical studies have demonstrated its efficacy in reducing Aβ burden, improving cognitive performance, and mitigating synaptic damage; however, challenges such as poor bioavailability, rapid metabolism, and limited blood–brain barrier penetration restrict its clinical applicability. Recent technological advances and selected modifications are being explored to overcome these limitations and enhance its therapeutic efficacy. This review summarizes the multifaceted neuroprotective mechanisms of RSV, the synergistic potential of natural compounds in enhancing neuroprotection, and the advancements in formulation strategies aimed at mitigating AD pathology. Leveraging the therapeutic potential of natural compounds represents a compelling paradigm shift for AD management, paving the way for future clinical applications.

## 1. Introduction

Alzheimer’s disease (AD) is a progressive neurodegenerative disorder characterized by cognitive decline, behavioral changes, and an inability to perform daily activities [1]. These symptoms impair the quality of life and increase dependency on caregivers. A hallmark feature of AD is the accumulation of amyloid-beta (Aβ) plaques in the brain, which disrupt neuronal communication, induce inflammation, and lead to neuronal death [2]. The failure to clear Aβ efficiently is considered to play a pivotal role in the onset and progression of the disease [3]. Despite the availability of drugs aimed at reducing Aβ levels, they have generally failed to prevent cognitive decline or halt disease progression [4,5,6].

Several studies have indicated a potential decline or stabilization in the prevalence and incidence of dementia over time in countries such as the United Kingdom and the United States. However, contrasting findings have emerged from Japan, where an increase in dementia prevalence has been reported. The reasons behind the observed decrease in age-specific dementia rates in some populations remain unclear. It is possible that improvements in education levels and public health efforts targeting cardiovascular health may play a role in reducing dementia risk. Recently, in Australia, a study investigating trends in dementia prevalence and survival among individuals receiving aged care services in Australia found that, between 2008 and 2014, the age- and sex-standardized prevalence of dementia among individuals in long-term care declined from 50.0% to 46.6%. Similarly, for those receiving home care, prevalence decreased from 25.9% in 2005 to 20.9% in 2014, an absolute change of −5.2 percentage points. This downward trend in dementia prevalence coincided with a decline in cerebrovascular disease among long-term care residents, despite rising rates of hypertension, diabetes, hypercholesterolemia, malnutrition, obesity, depression, and head injury. Notably, one-year mortality for individuals in long-term care remained stable over the study period [7]. Recently, F. E. Matthews et al. performed a two-decade comparison of dementia incidence in the Cognitive Function and Ageing Studies I and II, showing that age-specific incidence has declined, indicating that the annual number of new dementia cases has remained relatively constant [8].

The pathogenesis of AD is complex and involves a multifaceted interplay of genetic, molecular, and environmental factors. A hallmark feature of AD includes the accumulation of extracellular Aβ plaques and development of intracellular neurofibrillary tangles (NFTs). These pathological changes are accompanied by chronic neuroinflammation, mitochondrial dysfunction, oxidative stress, and disruption in the blood–brain barrier (BBB) [9]. The gut–brain axis (GBA) is a bidirectional communication network that links the gastrointestinal (GI) tract and the central nervous system (CNS). This intricate system involves neural, hormonal, immune, and metabolic pathways, which enable the gut and brain to influence each other. It is important for maintaining homeostasis and affects various physiological and psychological processes. GBA dysregulation is associated with conditions such as AD and causes mental health, anxiety, depression, and stress-related disorders that are linked to gut microbiota composition [10,11,12].

**Figure 1:**
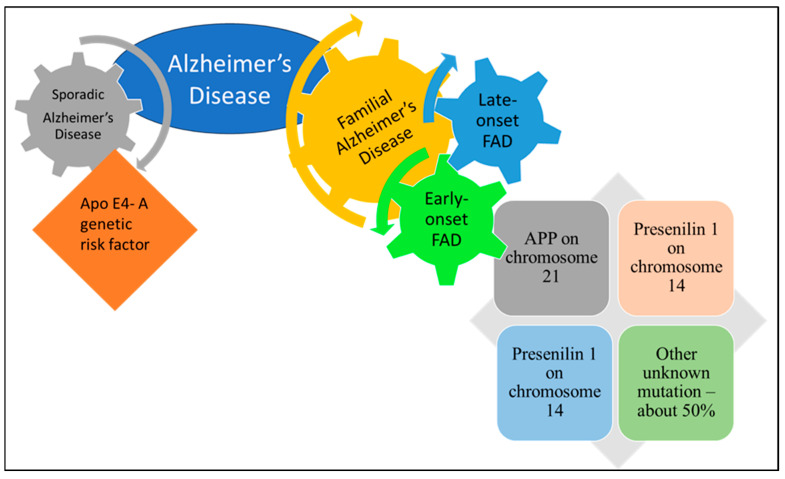
The classification of Alzheimer’s disease is categorized into familial Alzheimer’s disease (FAD) and sporadic Alzheimer’s disease. Each section highlights key features, such as prevalence, genetic factors, and pathology.

AD is the most common neurodegenerative disorder globally and may be classified into familial AD (FAD) and sporadic AD. FAD accounts for 1–5% of all cases and is inherited in an autosomal dominant manner. It typically presents as early-onset AD (EOAD) before the age of 65 [13]. This form is associated with genetic mutations in presenilin 1 (PSEN1), presenilin 2 (PSEN2), and the amyloid precursor protein (APP), which result in the abnormal production of Aβ peptide (Figure 1). These peptides aggregate to form plaques that are toxic to brain cells and contribute to the rapid progression of the disease. Mutations in these genes result in the abnormal production and accumulation of Aβ peptides, which form plaques that disrupt normal brain function. FAD is characterized by rapid progression with pronounced memory loss, cognitive impairment, and early neurological decline [14,15]. In contrast, sporadic AD represents approximately 95% of the cases and is categorized as late-onset AD (LOAD). It typically develops in individuals over 65 years of age. Although aging is the primary risk factor, various genetic, environmental, and lifestyle factors also contribute significantly. It is not directly inherited, although genetic predisposition and environmental factors may play a significant role. The APOE ε4 allele is a major genetic risk factor. The pathological mechanisms of LOAD include the abnormal processing of Aβ peptides, which leads to plaque formation, and the hyperphosphorylation of Tau proteins, which results in neurofibrillary tangles [16,17,18,19]. These processes contribute to neuronal dysfunction and death. Additional factors, such as synaptic and mitochondrial dysfunction, neurovascular impairments, oxidative stress, and chronic neuroinflammation, exacerbate AD. Although not directly inherited, advances in genome-wide association studies have identified numerous genetic polymorphisms associated with sporadic AD [20,21]. These polymorphisms are involved in pathways associated with Aβ processing, Tau protein metabolism, synaptic function, and neuroinflammatory responses. Among them, the APOE ε4 allele is considered the most significant genetic risk factor for LOAD. Collectively, these findings provide insight into the complex mechanisms underlying AD and open new avenues for the development of targeted therapies [22,23]. A list of physiological risk factors for AD is summarized in Table 1.

At the molecular level, the imbalance between Aβ production and clearance results in the formation of toxic oligomers, which impair synaptic function, activate microglia, and trigger a cascade of neuroinflammatory responses [24,25]. Tau pathology is driven by the hyperphosphorylation and misfolding of the Tau protein, which results in its aggregation into tangles that disrupt intracellular transport and contribute to neuronal death [26]. Mitochondrial dysfunction and oxidative stress exacerbate neuronal damage, whereas dysregulated calcium homeostasis and autophagy pathways contribute to disease progression [27]. Environmental factors, including lifestyle, diet, and exposure to toxins, also contribute to the disease by influencing these molecular and cellular mechanisms [28,29]. The intricate interdependence of these factors indicates the complexity of AD pathogenesis, complicating efforts to develop effective therapies. The treatment of AD faces numerous challenges because of its complexity and the limitations of current treatments. Therapeutic strategies for the prevention and treatment of AD encompass a range of approaches targeting various aspects of its progression and pathology [30]. Acetylcholinesterase (AChE) inhibitors, such as donepezil and rivastigmine, NMDA receptor antagonists, and memantine, are licensed for AD treatment; however, they provide limited symptomatic benefits [31]. In a recent study of Lecanemab, an anti-Aβ antibody showed that while it reduced amyloid markers in early AD, it resulted in only moderately ameliorated cognitive and functional decline compared with a placebo over 18 months [32]. In addition, studies into Tau-targeting therapies, such as antisense oligonucleotides aimed at reducing Tau protein levels, are ongoing, with early-phase trials exploring their potential efficacy [33]. Epidemiological studies indicate that NSAIDs, estrogen, HMG-CoA reductase inhibitors (statins), and tocopherol (vitamin E) may prevent AD; however, prospective randomized studies have yet to demonstrate their clinical efficacy. Significant advancements in molecular medicine continue to identify new drug targets for AD treatment [34]; however, the latest medications offer modest benefits in cognitive function and daily activities, and do not affect disease progression.

This ineffectiveness is attributed to various factors, including late-stage intervention, the involvement of other pathological mechanisms, such as Tau tangles and neuroinflammation, and the heterogeneity of AD [35]. Furthermore, the development of effective treatments has been hampered by inefficient drug delivery methods, particularly the BBB, which restricts the entry of therapeutic agents into the brain [36,37]. Current treatments primarily focus on symptomatic management and offer temporary relief for memory loss and confusion without addressing the underlying disease mechanisms. These conventional approaches have significant limitations as they are unlikely to slow disease progression. Despite over a century of research since AD was first identified, significant breakthroughs remain elusive, primarily because of the complexity of the disease, challenges in clinical trials, and lack of holistic approaches targeting multiple pathways. Advancing drug delivery systems and multitarget therapies may provide new opportunities for addressing this debilitating condition.

### 1.1. Challenges with the Currently Available Therapies

Aducanumab is a monoclonal antibody that targets Aβ plaques in the brain and was approved by the FDA in 2021 for early-stage AD. Although it marked a significant regulatory milestone, its use has sparked considerable debate because of issues surrounding its clinical benefits, costs, and safety [38]. Aducanumab can reduce amyloid plaques in the brain, which is a hallmark of AD; however, clinical trials have not provided conclusive evidence of its effectiveness in slowing cognitive decline. It was specifically approved for patients during the early stages of Alzheimer’s (mild cognitive impairment or mild dementia), with no established benefits for advanced cases [39]. Aducanumab is expensive, with annual treatment costs exceeding tens of thousands of dollars. This does not include the costs of monitoring, such as MRI scans, to detect adverse effects. Brain swelling (edema) and small brain bleeds, collectively termed Amyloid-Related Imaging Abnormalities (ARIAs), are the most common symptoms. These adverse effects typically resolve upon cessation of treatment but necessitate regular MRI monitoring, which adds to the burden on both patients and healthcare systems. The European Medical Agency did not approve aducanumab because of safety concerns and limited evidence of clinical efficacy. Aducanumab is administered through monthly intravenous infusions, which can be logistically challenging for patients, particularly those with mobility issues or limited access to infusion centers. Aducanumab has significant challenges that include its high cost, limited evidence of cognitive benefits, safety concerns, and logistical difficulties [40]. Furthermore, a recent study has reported that Biogen, the US drug company that developed Aducanumab (Aduhelm), has abandoned it [41].

Lecanemab has recently become the first disease-modifying therapy for AD to advance from accelerated to full FDA approval, following evidence demonstrating its clinical efficacy. This milestone supports the Aβ cascade hypothesis, which posits that Aβ aggregation and misfolding are central to the development of AD-related pathologies and cognitive decline. However, effectively delivering anti-amyloid monoclonal antibodies to the brain remains a major challenge. When administered intravenously or subcutaneously, only a minimal proportion—ranging from 0.01% to 0.11%—of these antibodies successfully cross the BBB and reach the CNS. This low brain penetration significantly impacts dose selection during clinical trials. A direct relationship exists between the dosage of anti-amyloid MABs and their ability to clear amyloid plaques and provide clinical benefits. Drugs such as aducanumab, lecanemab, donanemab, and gantenerumab have all shown dose-dependent effects. Nevertheless, higher doses are also linked to adverse events, most notably an increased incidence of ARIA. These safety concerns were key factors in the European Medicines Agency’s (EMA) decision to reject both aducanumab and lecanemab. Despite their proven capacity to reduce Aβ plaque burden, the modest cognitive improvements offered by these therapies were deemed insufficient to offset the associated ARIA risks [42].

Addressing these issues requires further clinical trials, economic analyses, and the collaborative efforts of researchers, regulators, and healthcare providers to improve treatment outcomes and accessibility [38].

### 1.2. BBB Limits Drug Delivery

The BBB prevents most drugs from crossing into the brain, which limits their effectiveness when targeting the CNS. More than 98% of small-molecule drugs and nearly 100% of large-molecule drugs do not cross the BBB [43]. BBB breakdown or dysfunction can occur before dementia, neurodegeneration, or brain atrophy, which enables cells, pathogens, and other harmful substances to enter the brain [44,45]. BBB is altered in AD; however, the mechanism is still not fully understood. This makes it difficult to develop therapeutics that target the BBB to delay its progression [46]. The BBB is an important part of the nervous system as it connects the CNS to the systemic circulation and other body systems. It is essential for neuronal function by limiting solutes that can enter the brain from the circulating blood [47]. BBB function is important for the early detection of AD and for studying its underlying pathology. Future studies should focus on the discovery and delivery of AD drugs and consider the BBB early in the drug discovery process [46].

### 1.3. Need for Precision Medicine

AD is the most common form of dementia in the elderly. It involves multifactorial pathology, including amyloid accumulation, vascular changes, systemic inflammation, genetic/epigenetic factors, and mitochondrial dysfunction. Despite extensive studies on neuroinflammation and amyloid-targeted therapies, current treatments have largely failed because of limitations in timing, patient selection, and the lack of a multitarget approach. The complete evaluation of such treatments requires robust biomarkers. Targeted anti-amyloidogenic and anti-inflammatory treatments should be tested in randomized, longitudinal, placebo-controlled studies using customized patient profiles (e.g., vascular or mitochondrial profiles). The widespread adoption and sharing of these methods will accelerate the development of innovative, personalized AD treatments with higher efficacy [48]. Personalized treatment based on genetic, environmental, and lifestyle factors is at an early stage of development [49,50,51]. A lack of comprehensive biomarkers to stratify patients and monitor disease progression has limited progress. Addressing these challenges requires interdisciplinary research, innovative therapeutic approaches, and a focus on prevention and early intervention.

### 1.4. Role of Natural Compounds in Neuroprotection

Natural compounds play a significant role in neuroprotection by mitigating the underlying mechanisms of neurodegenerative diseases. These compounds are often derived from plants, marine organisms, and microorganisms, and offer potential therapeutic strategies for neurological disorders, such as AD, PD, and Amyotrophic Lateral Sclerosis [52]. Of these, natural phenolic compounds have attracted considerable attention because of their diverse biological activities and therapeutic benefits for human health. Polyphenols (phenolic acids, flavonoids, stilbenes, and coumarins) play an important role in neuroprotection by modulating cell function and attenuating oxidative stress, inflammation, and apoptosis in animal models [53]. Antioxidants have emerged as promising compounds in preclinical studies for combating neurodegeneration. However, they have not produced significant effects in clinical trials in over a decade [54]. Vitamins, such as A, E, and C, along with polyphenolic compounds, such as flavonoids, have excellent antioxidant properties. These antioxidants are predominantly obtained through the diet, whereas medicinal herbs are also rich sources of flavonoids. By preventing oxidative stress (ROS)-induced neuronal damage, antioxidants play a protective role in mitigating the effects of oxidative stress and promoting neuronal health in neurodegenerative conditions. Natural compounds, such as flavonoids, polyphenols, and vitamins, scavenge free radicals and reduce oxidative damage to neural tissues [55].

**Figure 2:**
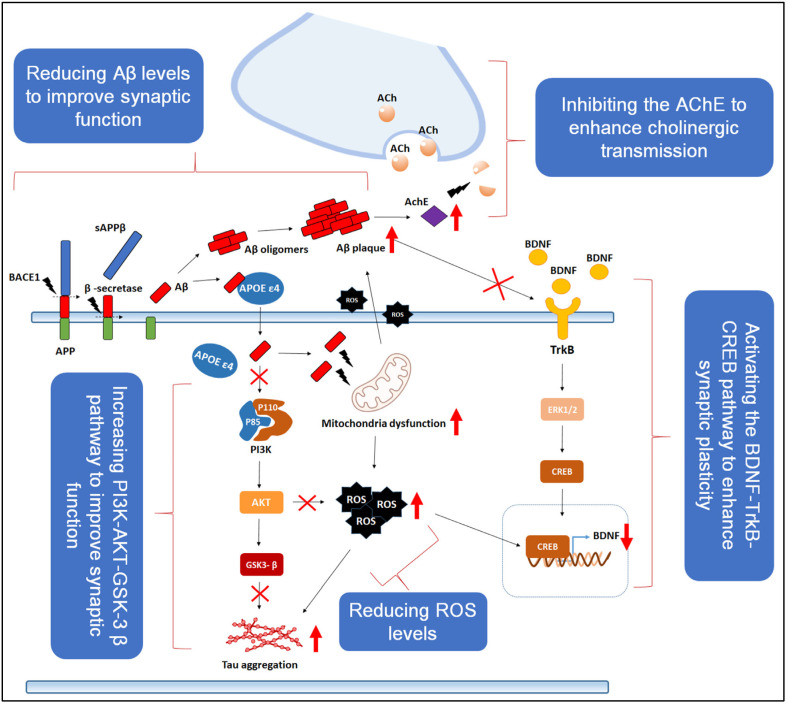
Multifaceted neuroprotective actions of a natural compound mediated through antioxidant activity, cholinesterase inhibition, and modulation of key signaling pathways. The figure is adopted from [56]. (Black arrows denote the sequential flow of processes or signaling pathways, and red upward arrows indicate an increase in expression, activity, or accumulation of specific molecules or cellular responses, whereas red downward arrows indicate a decrease).

Natural compounds with recognized neuroprotective properties often act through a variety of interconnected mechanisms. These include the inhibition of acetylcholinesterase (AChE), which enhances cholinergic signaling, as well as the reduction of Aβ accumulation and tau hyperphosphorylation. Activation of the PI3K-AKT-GSK-3β signaling cascade further supports synaptic integrity and neuronal survival. In addition, the antioxidant and anti-inflammatory activities of such compounds help counteract neurotoxicity and cellular stress. They may also stimulate the BDNF-TrkB-CREB pathway, which is vital for promoting synaptic plasticity and cognitive function. Moreover, these natural agents can mitigate synaptic impairment induced by ROS and oxidative damage [56] (Figure 2).

Resveratrol (RSV) is a polyphenol used in pharmaceuticals for its antioxidant, anti-inflammatory, and cardioprotective effects. It is also added to cosmetics for anti-aging and skin protection, and in nutraceuticals as a dietary supplement to improve health [57]. The role and application of RSV in AD management and future perspectives in AD therapeutics are further discussed below.

### 1.5. Resveratrol (RSV)

**Figure 3:**
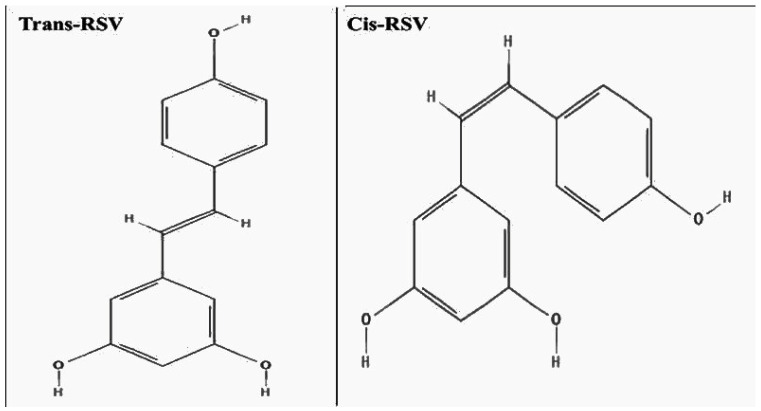
Trans and cis forms of RSV. Trans-RSV has a linear configuration because of the trans-orientation of the double bond between the two phenol rings. Cis-RSV adopts a bent structure because of the cis-orientation of the double bond.

RSV is also known as 3,5,4′-trihydroxystilbene with a molecular weight of 228.25 g/mol. It is a natural polyphenol that contains two phenol rings connected by a double styrene bond. RSV is present in isomeric forms, cis and trans, as shown in Figure 3. The trans-isoform is more stable, bioactive, and is the naturally abundant form [58]. Its chemical structure features three hydroxyl (-OH) groups, which are required for its biological activity. These hydroxyl groups enable the molecule to scavenge free radicals, chelate metals, and interact with macromolecules, thus contributing to its antioxidative, antimicrobial, and health-promoting properties [59,60,61]. Presence of -OH group also contributes to the compound’s ability to influence inflammatory signaling and provide neuroprotective effects. The positioning and number of these phenolic groups are critical to the compound’s biological activity, as structural modifications can markedly affect both its functional potency and metabolic stability. As a result, many studies on resveratrol analogs aim to modify these functional sites to improve pharmacokinetic properties and therapeutic efficacy, supporting the development of more potent and bioavailable resveratrol-based agents [62]. An experimental study suggested that the 4′-OH group of RSV plays a crucial role in its direct interaction and activation of PPARα [63].

RSV is a low-molecular-weight polyphenolic compound that belongs to the solenoid family, which is characterized by hydroxylated derivatives of stilbene. It is present in various plants, including grapes, berries, peanuts, and red wine [64]. It acts as a phytoalexin, a protective antimicrobial substance synthesized by plants in response to stressors, such as pathogens, UV radiation, or environmental damage. When plants are exposed to bacteria or fungi, such as *Botrytis cinerea*, they produce RSV to inhibit the growth and reproduction of these microorganisms, thereby safeguarding themselves against infection. The structure of RSV imparts strong antioxidant properties that neutralize free radicals and protect plant cells from oxidative stress. In addition to its role in plant defense, RSV has attracted attention for its potential health benefits in humans. Thus, RSV serves as a vital plant defense molecule and a compound of interest for human health [65].

The anti-inflammatory effects of RSV further contribute to its potential in managing chronic inflammatory conditions. The synthesis of RSV may be categorized as plant extraction, biosynthesis, and chemical synthesis. Although plant extraction remains a common method, it is constrained by factors such as low yield, plant growth habits, and low extraction efficiency. Despite these limitations, some companies continue to extract trans-RSV with varying levels of purity, primarily from the root extracts of *Polygonum multiflorum* [66]. *Polygonum multiflorum* contains stilbene compounds, such as RSV and THSG (tetrahydroxystilbene glucoside) [67], and is valued for its anti-aging and nerve regeneration properties, which contribute to its potential role in AD treatment [68]. *Pinus pinaster* (Maritime Pine), *Pinus sylvestris* (Scots Pine), and *Pinus taeda* (Loblolly Pine) all contain RSV and related stilbenoids, primarily in their bark, which contribute to their antioxidant and anti-inflammatory properties. The bioactive trans form of RSV is abundant in *Arachis hypogaea* and its plant parts, with levels enhanced by external stimuli, such as UV light and stress hormones. The RSV synthase genes (RS1–RS4) in peanuts are linked to increased RSV production, making peanuts a promising nutraceutical. UV and ultrasound (US) treatments increase RSV, piceid (glucoside form of RSV), and total stilbenes in peanuts, but reduce sensory acceptance, with the US inducing higher stilbene levels compared with UV. Three to five peanut bars containing US-treated peanuts can match the RSV content of a 140 mL glass of red wine [69,70]. *Vaccinium berries*, including blueberries, cranberries, and lingonberries, contain RSV, pterostilbene, and piceatannol, with lingonberries having the highest RSV levels when compared with grapes. These stilbenes are known for their antioxidant and cancer-preventive properties and enhance the health benefits of these fruits [71].

### 1.6. Pharmacokinetics and Bioavailability

RSV exhibits a variety of pharmacological properties, but its use is limited because of low bioavailability, which is influenced by metabolic enzymes, such as cytochrome P450s, UDP-glucuronosyltransferases, and sulfotransferase. Over 20 metabolites of RSV have been identified, which interact with these enzymes [72]. RSV and extra virgin olive oil (EVOO) have complementary antioxidant, anti-inflammatory, and neuroprotective properties that promote longevity and cognitive health, thereby reducing the risk of age-related diseases. Their combined consumption may enhance their bioavailability and efficacy, offering significant benefits for aging populations [73]. EVOO may aid in clearing toxic proteins, such as Aβ and Tau, from the brain, while reducing neuroinflammation and oxidative stress, which are key factors in AD progression. Its neuroprotective properties support cognitive function and may help slow age-related decline [74].

## 2. Neuroprotective Mechanisms of RSV

### 2.1. Anti-Inflammatory and Antioxidant Properties

**Figure 4:**
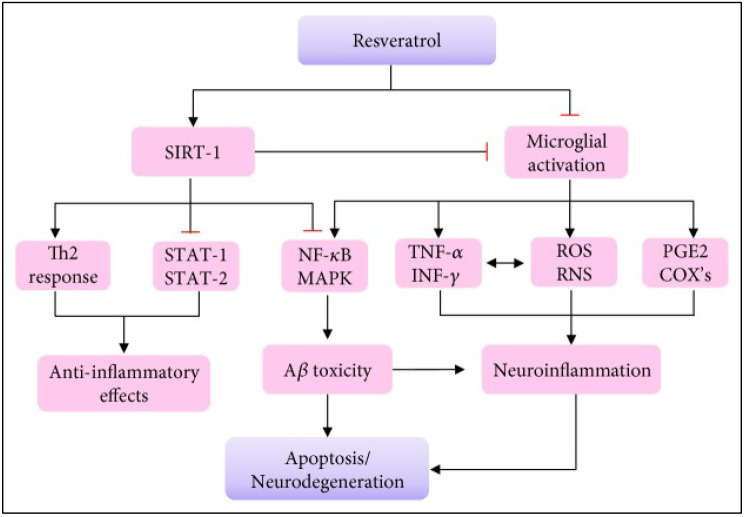
Schematic illustrating the proposed mechanisms through which resveratrol exerts its neuroprotective effects. Resveratrol activates SIRT-1, which promotes a Th2 immune response and exerts anti-inflammatory effects, while inhibiting the STAT-1/STAT-2 and NF-κB/MAPK signaling pathways. This reduces Aβ toxicity and apoptosis/neurodegeneration. In addition, resveratrol suppresses microglial activation, leading to reduced production of proinflammatory mediators, such as TNF-α, IFN-γ, ROS, RNS, PGE2, and COX enzymes. This downregulation ultimately reduces neuroinflammation and its downstream effects, including neuronal apoptosis and degeneration. This figure is adopted from [76].

The anti-inflammatory and antioxidant effects of RSV contribute to neuronal protection by reducing oxidative stress and inflammation. RSV exerts strong antioxidant properties and ameliorates oxidative stress, which damages neurons in AD [75]. It activates silent information regulator-1 (SIRT1), which promotes the growth and differentiation of neurons. RSV inhibits neuronal death by repressing the activity of p53, a protein linked to cell death. RSV reduces the toxicity of Aβ peptides, promotes the formation of new neurons, and protects the hippocampus from damage. In addition, it suppresses the activation of M1 microglia, which are immune cells that trigger inflammation and neurodegeneration. It also increases the production of Th2 cytokines, which are anti-inflammatory molecules, by enhancing SIRT1 activity. RSV shows promise in protecting neurons and slowing the progression of AD by reducing Aβ aggregation, oxidative stress, and inflammation, with a particular focus on its interaction with SIRT1 (Figure 4) [76,77].

Monomeric C-reactive protein (mCRP) is a proinflammatory molecule linked to an increased risk of AD following stroke. RSV protects brain cells from the harmful effects of mCRP. In one study, BV2 cells derived from mouse microglia were exposed to mCRP for 24 h, with and without RSV treatment. mCRP (50 µg/mL) activated inflammatory pathways, such as the nitric oxide and NLRP3 inflammasome pathways, increased cyclooxygenase-2 activity, and induced the release of proinflammatory cytokines. These changes indicate a heightened inflammatory state, which contributes to the progression of AD. RSV ameliorated these effects by inhibiting the inflammatory changes and boosting antioxidant defenses. It increased the expression of the antioxidant enzymes Cat (catalase) and Sod2 (superoxide dismutase 2), both of which help neutralize oxidative stress. RSV also activated key regulatory genes, including Sirt1 (involved in cellular stress resistance and longevity) and Nfe2l2 (a major regulator of antioxidant responses). Importantly, RSV prevented the nuclear translocation of NF-ĸB, which drives inflammation. The study also confirmed the protective effects of RSV in primary glial cell cultures, showing its broader applicability. By reducing inflammation and oxidative stress, RSV mitigates the harmful effects of proinflammatory agents, such as mCRP, which suggests a therapeutic approach for preventing AD [75]. Extensive in vitro and in vivo studies have demonstrated these effects; however, low bioavailability limits its efficacy and has prompted efforts to improve its delivery [78]. A Phase I clinical study by Boocock et al. [79] investigated the pharmacokinetics of oral trans-RSV in healthy volunteers who received single doses ranging from 0.5 g to 5 g. Despite high oral intake, the study found that peak plasma concentrations of unmetabolized (free) RSV remained low, ranging from only 0.3 μM to 2.4 μM. This is markedly below the concentrations typically required to exert biological activity in vitro, which often exceed 10 μM. The limited systemic availability was attributed to extensive first-pass metabolism, with glucuronide and sulfate conjugates of resveratrol detected at concentrations 20- to 40-fold higher than the parent compound. These findings demonstrate that while RSV is efficiently absorbed, its oral bioavailability is severely compromised by rapid metabolic conversion, underscoring the need for improved delivery strategies to achieve therapeutically relevant concentrations in vivo [79].

According to Capiralla’s group, RSV is associated with anti-inflammatory effects and is currently under investigation in clinical trials for AD [80]. It prevents the activation of murine RAW 264.7 macrophages and microglial BV-2 cells treated with the TLR4 ligand, lipopolysaccharide (LPS). RSV preferentially inhibits activation of nuclear factor j-light-chain-enhancer of activated B cells (NF-jB) following LPS stimulation by interfering with IKK and IjB phosphorylation, an effect that potently reduces the transcriptional stimulation of NF-jB target genes, such as tumor necrosis factor-a and interleukin-6. Consequently, the downstream phosphorylation of signal transducer and activator of transcription STAT1 and STAT3 upon LPS stimulation is also inhibited by RSV. Capiralla et al. found that RSV acts upstream in the activation cascade by interfering with TLR4 oligomerization following receptor stimulation. RSV treatment also prevented the proinflammatory effects of fibrillar Ab on macrophages by inhibiting the effect of Aβ on IjB phosphorylation, activation of STAT1 and STAT3, and the secretion of tumor necrosis factor-α and interleukin-6. Orally administered resveratrol in a mouse model of cerebral amyloid deposition lowered microglial activation associated with cortical amyloid plaque formation. Taken together, these studies provide strong evidence that RSV exerts in vitro and in vivo anti-inflammatory effects against Aβ-triggered microglial activation. Further studies in cell culture systems indicate that RSV acts through a mechanism involving the TLR4/NF-jB/STAT signaling cascade [80].

### 2.2. Modulation of Amyloid-Beta (Aβ) Accumulation

**Figure 5:**
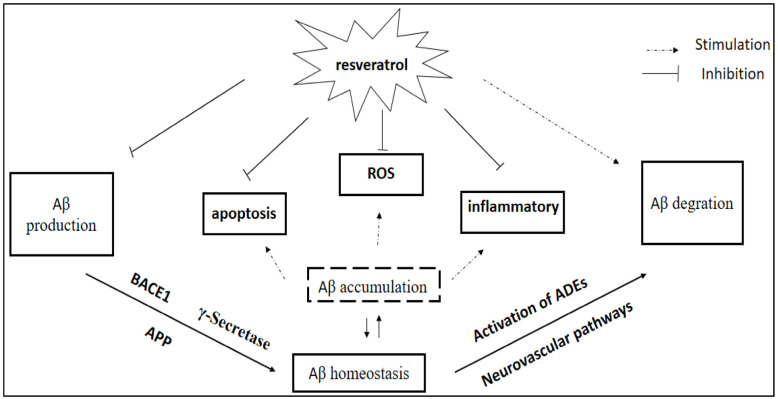
Resveratrol’s influence on amyloid-β (Aβ) homeostasis. Resveratrol modulates key processes involved in Aβ metabolism by reducing its production and enhancing its degradation. It inhibits the generation of reactive oxygen species (ROS), apoptosis, and inflammatory responses, while promoting the activity of Aβ-degrading enzymes (ADEs) and maintaining Aβ balance. APP: amyloid precursor protein; BACE: β-site APP-cleaving enzyme; ROS: reactive oxygen species; ADEs: Aβ-degrading enzymes. The figure is adopted from [85].

RSV influences reduction in Aβ accumulation, which is important in AD pathology. Recent in vitro and in vivo studies have explored the role of RSV in AD (Figure 5). Despite promising effects, its clinical application is limited due to its low bioavailability [81].

Aβ is a key constituent of senile plaques, the characteristic lesions commonly observed in the neocortex and hippocampus of AD brains. Overproduction of the highly insoluble 42-amino-acid Aβ42 peptide is closely associated with mutations in the three genes implicated in early-onset autosomal dominant familial AD. Ge et al. (2012) [82] studied how RSV interacts with Aβ proteins and found that RSV can directly bind to both the single (monomer) and aggregated (fibril) forms of Aβ (1–40 and 1–42). RSV exhibited stronger binding to the monomer Aβ (1–40), but stronger binding to fibril Aβ (1–42). Compared with Congo red, RSV exhibited superior binding to the monomers, but not to the fibrils, and showed a weaker binding strength overall. When RSV was mixed with Aβ, it reduced the amount and size of the harmful fibrils. It also stained brain plaques in Alzheimer’s patients, showing that it interacts with Aβ in the brain [82]. RSV does not reduce Aβ production because it does not affect β- or γ-secretase activity. Instead, it promotes the breakdown of Aβ inside the cells through proteasome-dependent degradation. This effect is blocked by proteasome inhibitors or by silencing the β5 proteasome subunit, thus confirming the pathway [83]. RSV protects PC12 cells from Aβ25–35-induced neurotoxicity by enhancing autophagy, as evidenced by increased LC3-II expression and autophagosome formation. This protective effect is dependent on the activation of the TyrRS-PARP1-SIRT1 signaling pathway. Blocking autophagy or inhibiting TyrRS, PARP1, or SIRT1 significantly reduces its neuroprotective effects [84].

### 2.3. Reduction in Tau Protein Hyperphosphorylation

The primary Tau protein phosphatase in the brain is protein phosphatase 2A (PP2A), whereas the primary Tau protein kinase is glycogen synthase kinase-3β (GSK3β). Reduced PP2A expression and activity and/or overactivation of GSK3β have been reported during the onset and progression of AD. By decreasing Tau hyperphosphorylation, RSV may mitigate neurofibrillary tangle formation, which is a hallmark of AD. Studies in various species have identified the role of RSV in Tau protein dephosphorylation. In one study, the role of RSV was examined in an AD rat model. RSV protected the rats from cadmium chloride-induced memory loss and Tau protein hyperphosphorylation. RSV improved memory, increased glutathione levels, and inhibited malondialdehyde-induced ROS levels. RSV also increased levels of p-GSK3β and p-PP2A and activated the PI3K/Akt signaling pathway [86].

RVS reduces Tau protein hyperphosphorylation by activating protein phosphatase 2A (PP2A) through the disruption of the MID1-PP2A complex. It increases PP2A activity by lowering MID1 expression, which normally promotes the degradation of its catalytic subunit [87]. In in vitro FA-treated N2a cells, RSV reduced cytotoxicity, apoptosis, and Tau hyperphosphorylation by inhibiting GSK-3β and CaMKII, which are key enzymes in Tau modification. Taken together, these results suggest that RSV protects against Tau-related Alzheimer’s pathology by promoting dephosphorylation and preventing harmful protein changes [88]. However, the clinical application of RSV remains limited because of its low bioavailability, prompting efforts to enhance its efficacy [78].

### 2.4. Influence on Key Signaling Pathways

RSV affects PI3K/Akt, SIRT1, and Wnt, which are involved in cell survival, aging, and neuroprotection. RSV enhances hippocampal neurogenesis by activating SIRT1, which in turn, positively regulates the Wnt signaling pathway [89]. The therapeutic effects of RSV in AD may involve the suppression of the PI3K signaling pathway. Key targets include MAPK1, HRAS, EGFR, and MAP2K1, which indicate potential interactions with other therapeutic agents targeting these pathways. A recent study analyzed the efficacy of RSV in AD through a meta-analysis of five clinical trials encompassing 271 patients. RSV improved ADAS-ADL scores and increased CSF and plasma Aβ40 levels compared with a placebo; however, no significant changes were observed in the MMSE scores, Aβ42 levels, or brain volume [90]. Adverse effects were comparable between the two groups. Network pharmacology identified the PI3K signaling pathway as a primary mechanism, with key targets including MAPK1, HRAS, EGFR, and MAP2K1 [90]. RSV attenuates Aβ25–35-induced neurotoxicity by inducing autophagy through the TyrRS-PARP1-SIRT1 signaling pathway [84]. Feng et al. [91] examined the protection of RSV against the neurotoxicity of Aβ25–35 and further explored its underlying mechanism. PC12 cells were injured with Aβ25–35, and resveratrol was added at various concentrations to the culture medium. RSV increased cell proliferation and reduced apoptosis. Moreover, it stabilized intercellular Ca^2+^ homeostasis and attenuated Aβ25–35 neurotoxicity. In addition, Aβ25–35-suppressed SIRT1 activity, which was significantly reversed by RSV, resulting in the downregulation of Rho-associated kinase 1 (ROCK1). As a downstream signal molecule, ROCK1 was negatively regulated by SIRT1. Taken together, this study demonstrated that the SIRT1-ROCK1 pathway plays an important role in the molecular pathology of AD [91].

The multitarget-directed ligand strategy offers a promising approach to the traditional one-drug–one-target approach. RSV is known for its beneficial effects against AD and has been used as a pharmacophore for designing new derivatives. Of these, compound 6r showed moderate cholinesterase inhibition (AChE IC_50_ = 6.55 μM; BuChE IC_50_ = 8.04 μM), significant inhibition of Aβ42 aggregation (57.78% at 20 μM), and inhibitory activity against monoamine oxidases (MAO-A IC_50_ = 17.58 μM; MAO-B IC_50_ = 12.19 μM). This compound has emerged as a balanced potential anti-Alzheimer agent and provides a foundation for the further development of multitarget-directed RSV derivatives for AD therapy [92].

## 3. RSV in Preclinical and Clinical Studies for AD

Several neuroprotective activities have been discussed above showing the potential role of RSV in preclinical and clinical studies. A series of novel RSV surrogate molecules was developed and examined for their potential as multifunctional agents to combat AD. These molecules were designed to inhibit acetylcholinesterase (AChE) and butyrylcholinesterase (BChE), which are enzymes implicated in AD, while also demonstrating antioxidant properties. The synthesis involved the reaction of (E)-4-(3,5-Dimethoxystyryl) aniline with benzaldehyde and its derivatives using ethanol and glacial acetic acid, using the Schiff base formation method. Six compounds (RSM1–RSM6) were synthesized and characterized by FT-IR, ^1^H-NMR, ^13^C-NMR, and mass spectrometry to confirm their structural integrity. Of these, RSM5 exhibited the most potent dual inhibitory activity against AChE and BChE, which is essential for mitigating AD symptoms. In addition, RSM5 showed minimal cytotoxicity and exhibited significant antioxidant activity. These attributes highlight RSM5 as a promising lead molecule for AD drug development, as it combines safety, efficacy, and multifunctional therapeutic potential. Six RSV surrogate molecules were synthesized and characterized as potential anti-Alzheimer’s agents. Of these, RSM5 was superior with its potent dual cholinesterase inhibition, minimal cytotoxicity, and strong antioxidant activity, making it a promising lead for future AD drug development [93]. AD affects approximately 40 million individuals worldwide, with cases expected to triple over the next 50 years because of an aging population and a lack of effective treatments. Three caffeic acid–RSV hybrid derivatives (compounds **1**–**4**) showed superior inhibition of the key AD enzymes, beta amyloid cleaving enzyme 1 (BACE1) and AChE, compared with the parent compounds and standard drugs. Compound **4** showed strong BACE1 inhibition (IC_50_ = 69 nM), and compound **3** exhibited strong AChE inhibition (IC_50_ = 1.93 μM), which was supported by molecular docking, and highlighted their potential as anti-AD therapeutics [94]. Olesoxime-RSV (OLX-RSV) encapsulated in exosomes was developed as a potential AD treatment. The nanocomposite inhibited Aβ1-42 aggregation, crossed the BBB safely, and showed good biocompatibility in SHSY5Y cells. It reduced apoptosis, enhanced antioxidant defenses, and improved learning and memory in APP/PS1 mice. These results highlight OLX-RSV-loaded exosomes as a promising strategy for AD treatment [95]. A 52-week phase 2 trial evaluated the safety, tolerability, and effects of RSV in 119 participants with mild to moderate AD. They received a placebo or RSV with dose escalation up to 1000 mg twice daily. Biomarkers (CSF and plasma Aβ40, Aβ42, Tau, and phospho-Tau 181) and MRI brain volume were assessed at baseline and following treatment. RSV and its metabolites were detectable in the plasma and CSF, indicating CNS penetration. Adverse events included nausea, diarrhea, and weight loss. CSF and plasma Aβ40 levels decreased less in the RSV group; however, brain volume loss was higher compared with that in the placebo group. Overall, RSV was safe and well tolerated [96]. Table 2 and Table 3 summarize the potential application of RSV as a therapeutic for AD treatment in in vitro and in vivo studies, respectively.

## 4. Future Perspectives and Research Directions

AD is a progressive neurodegenerative disorder characterized by Aβ accumulation, which leads to brain damage and dementia. Aβ oligomers formed through the catalytic degradation of APP by BACE1, resulting in insoluble plaques that disrupt mitochondrial function and cause neuronal damage. Targeting Aβ pathways has been a focus in the development of AD treatments; however, many drugs cannot cross the BBB, which limits their effectiveness. Advances in nanotechnology offer promising solutions for targeted drug delivery by combining natural products with nanomedicine to create more effective AD therapies [111]. Despite its therapeutic benefits, RSV is limited because of its poor bioavailability and extensive metabolism, thus requiring large doses for effectiveness. Derivatization techniques, such as hydroxylation and glycosylation, can improve its bioavailability and therapeutic outcomes. Encapsulating RSV in nanoparticles, such as polymers and solid lipids, enhances its solubility and absorption and increases its efficacy. These approaches may be useful for developing more effective RSV-based treatments [112].

Conflicting results from clinical trials have resulted, in part, from variations in dosing protocols. Efforts to improve bioavailability include combining RSV with foods or phytochemicals, micronization, controlled release systems, and nanotechnology. Although laboratory studies suggest that these methods may optimize RSV efficacy, human data remain limited. Further studies are needed to better understand and enhance its bioavailability for clinical use, as preclinical evidence supports these strategies. Limited human data exist on RSV bioavailability, necessitating further exploration for clinical optimization [113].

Recent studies have highlighted the potential of RSV, a naturally occurring polyphenol, as a therapeutic agent for AD because of its neuroprotective properties [114]. Clinical trials suggest that RSV may slow cognitive decline in AD patients, although more clinical studies are needed to confirm its efficacy and safety [115]. A list of clinical trials of resveratrol for AD management is summarized in Table 4. RSV also interacts with key molecular pathways, including the activation of SIRT1 and modulation of Aβmetabolism, which are important to AD pathogenesis. Future studies should focus on optimizing dosage, understanding long-term effects, elucidating mechanisms, and exploring combination therapies to maximize their therapeutic potential. AD is characterized by Aβ peptide accumulation, Tau protein phosphorylation, oxidative stress, and inflammation, which leads to cognitive decline. RSV shows neuroprotective, anti-inflammatory, and antioxidant properties, reducing Aβ aggregation and hippocampal degeneration, while promoting neurogenesis through SIRT1 activation. Its potential as a therapeutic agent for AD is promising for the management of other neurodegenerative disorders [97]. A systematic review of randomized controlled trials (RCTs) assessed its effects on cognitive and functional performance in AD patients, with four RCTs showing potential benefits in delaying cognitive decline. Despite limited human studies, RSV appears to slow AD progression compared with placebo treatment [97].

In neurological diseases, such as Alzheimer’s and Parkinson’s disease, RSV protects neurons from oxidative damage, toxicity, and apoptosis. It also induces apoptosis in brain cancer cells while inhibiting angiogenesis and tumor invasion. Despite its therapeutic potential, RSV has poor water solubility, chemical instability, and low bioavailability, which limit its benefits. Nanotechnology offers solutions by encapsulating RSV in nanocarriers, such as liposomes and lipid or polymeric nanoparticles, to enhance stability, prolong half-life, and improve brain targeting. These advances in nanomedicine may overcome the limitations of RSV, improving its efficacy for treating neurological diseases [118]. Nanotechnology-based delivery systems have been developed to enhance RSV effectiveness. These nanocarriers, designed for intranasal, oral, or parenteral administration, have demonstrated success in various pharmacological, pharmacokinetic, and cell studies, thus enhancing brain delivery and stability. Although human clinical trials with nanocarriers are lacking, studies in animal and cell models indicate promising therapeutic effects for neurological diseases using RSV-loaded nanosystems [119].

AD is a progressive neurodegenerative disorder that severely impacts memory and cognitive function and affects millions of individuals worldwide. Existing treatments primarily focus on symptom management, with no approved therapies to modify the underlying disease mechanisms. Gene therapy has emerged as a promising strategy to target the pathophysiology of AD and potentially provide disease-modifying treatments [120]. Many drugs that have shown promise in preclinical studies have failed in clinical trials because of insufficient efficacy or adverse effects. Challenges in patient selection and the inability to stratify subgroups with distinct pathological mechanisms have contributed to trial failures. The withdrawal of drugs, such as thiethylperazine, CT1812, Crenezumab, CNP520, and Lecanemab, highlights the need for rigorous preclinical evaluations to improve success rates and reduce costly setbacks.

These limitations underscore the importance of rigorous preclinical assessments to predict clinical outcomes more accurately, thereby preventing the premature initiation of high-risk clinical trials. This approach may save valuable time, money, and effort, and pave the way for more effective strategies for AD drug development [121]. Mucoadhesive nanoemulsions are an advanced drug delivery system designed to enhance bioavailability and prolong the retention of therapeutic agents at the mucosal surfaces. These formulations combine nanoemulsions (stable, nanosized oil-in-water or water-in-oil dispersions) with mucoadhesive properties. The combination of curcumin and resveratrol in mucoadhesive nanoemulsions is a promising strategy for enhanced drug delivery in neurodegenerative diseases, including AD. This approach improves the bioavailability, sustained release, and targeted drug delivery to the brain [122].

## 5. Conclusions

The therapeutic potential of RSV in AD lies in its neuroprotective properties, which may overcome the pathological features of this disease, such as Aβ accumulation, oxidative stress, and inflammation. The ability of RSV to modulate various molecular pathways, including the activation of SIRT1 and downregulation of CD147, plays an important role in reducing Aβ production and secretion. By enhancing Aβ clearance, improving mitochondrial function, and reducing oxidative damage, RSV may preserve neuronal health and function. In addition, its anti-inflammatory effects may mitigate neuroinflammation, a significant contributor to AD progression. The evidence from both in vitro and in vivo studies supports the role of RSV in slowing AD progression and its potential as a therapeutic agent. In conclusion, RSV offers promise in developing AD treatments, focusing on its ability to modulate neurodegenerative processes and promote brain health. Further clinical studies are needed to translate these findings into effective therapies for AD.

## Figures and Tables

**Table 1:** Physiological risk factors for Alzheimer’s disease.

Category	Risk Factor	Mechanism/Pathophysiology
Amyloid Pathology	Amyloid-β (Aβ) accumulation	Overproduction or impaired clearance of Aβ peptides, leading to plaque formation and synaptotoxicity.
Tau Pathology	Hyperphosphorylation of tau	Formation of neurofibrillary tangles; disrupted microtubule stability and axonal transport.
Genetic Factors	APOE ε4 allele	Impairs Aβ clearance, promotes lipid dysregulation and neuroinflammation.
	APP, PSEN1, PSEN2 mutations	Increase Aβ42 production via altered γ-secretase activity.
Neuroinflammation	Chronic glial activation	Microglia and astrocytes release proinflammatory cytokines (e.g., IL-1β, TNF-α); NLRP3 inflammasome activation.
Oxidative Stress	ROS overproduction	Mitochondrial damage and lipid, protein, DNA oxidation contribute to neuronal death.
Mitochondrial Dysfunction	Impaired ATP production	Reduced energy metabolism, increased ROS, and cytochrome c release.
Synaptic Dysfunction	Aβ oligomers	Disrupt synaptic transmission, inhibit LTP, and cause early synapse loss.
Proteostasis Impairment	UPS and autophagy dysfunction	Accumulation of misfolded proteins; reduced degradation of Aβ and tau aggregates.
BBB Dysfunction	Reduced clearance and barrier integrity	Impaired Aβ efflux, increased neurotoxin and immune cell entry into CNS.
Lipid Metabolism Dysregulation	Altered cholesterol transport	Affects APP processing and tau phosphorylation; APOE isoform-specific effects.
Insulin Resistance	“Type 3 diabetes”	Impaired PI3K/Akt signaling; decreased glucose uptake and neuroprotection.
Excitotoxicity	Excess glutamate/NMDA activation	Calcium overload, mitochondrial dysfunction, and neuronal death.
Calcium Dyshomeostasis	Disrupted Ca^2+^ signaling	Affects mitochondrial integrity, activates cell death pathways.
Epigenetic Changes	DNA methylation, histone modifications	Alters gene expression relevant to inflammation, metabolism, and synaptic function.
Vascular Dysfunction	Cerebral hypoperfusion	Reduced oxygen/nutrient delivery; promotes white matter lesions and Aβ retention.
Gut–Brain Axis Disruption	Microbiota imbalance	Increases systemic and CNS inflammation; affects BBB and amyloid pathology.

**Table 2:** Summary of in vitro studies that provide strong evidence for the neuroprotective role of RSV against the pathological features of AD.

RSV Source	Cell Model	Key Findings	References
Synthetic	H19–7 hippocampal neuronal cells	Resveratrol protected against β-amyloid-induced oxidative damage and preserved memory-associated proteins	[97]
Synthetic	murine neuroblastoma (N2A) cell model	Demonstrated the anti-Alzheimer effects of resveratrol and curcuminoids, highlighting inhibition of Aβ aggregation	[98]
Natural	BV2 microglial cells	Resveratrol inhibited LPS and mCRP-induced COX-2 expression, reduced proinflammatory cytokine release, and upregulated antioxidant enzymes, suggesting neuroprotective effects against AD-related inflammation	[75]
Synthetic	SH-SY5Y neuroblastoma cells	Resveratrol activated AMPK signaling and modulated amyloid-β peptide metabolism	[99]
Synthetic	SH-SY5Y neuroblastoma cells	Olesoxime and resveratrol co-encapsulated in exosomes suppressed Aβ_1_–_42_ aggregation, protected against Aβ-induced cytotoxicity, and enhanced antioxidant defenses, indicating potential therapeutic synergy in AD models	[95]
Synthetic	Dopaminergic neurons	Resveratrol promoted the astroglial release of BDNF and GDNF, offering neurotrophic support	[100]
Synthetic	SH-SY5Y neuroblastoma cells	Inhibited β-amyloid oligomeric cytotoxicity without preventing oligomer formation	[101]
Synthetic	SK-N-BE neuroblastoma cells	Showed antioxidant effects and protection against α-synuclein and Aβ42 toxicity	[102]
Natural	Primary rat microglia	Resveratrol reduced prostaglandin E2 production and free radical formation in activated microglia	[103]

**Table 3:** In vivo studies demonstrating the therapeutic potential of RSV in AD models.

Source	Animal Model	Key Findings	References
Synthetic	Colchicine-induced AD in Wistar rats	Resveratrol (RS) at 10 mg/kg, both alone and combined with donepezil (DPZ), significantly reduced β-amyloid plaques and neurofibrillary tangles in the hippocampus. Prophylactic administration of RS exhibited neuroprotective effects, with the combination therapy yielding the most pronounced benefits	[104]
Synthetic	Streptozotocin-induced AD in rats	Oxyresveratrol-β-cyclodextrin complex improved cognitive function and reduced histone deacetylase activity in the hippocampus and frontal cortex. The treatment also decreased malondialdehyde levels, indicating reduced oxidative stress	[105]
Synthetic	3xTg-AD mice	Intranasal administration of resveratrol nanoparticles protected mice against retinal and brain neurodegeneration. Treatment reduced amyloid-beta and phosphorylated Tau deposition in the brain, suggesting the potential for noninvasive delivery	[106]
Synthetic	APP/PS1 transgenic mice	Exosomes co-encapsulating olesoxime and resveratrol suppressed amyloid-beta aggregation, enhanced antioxidant defenses, and improved spatial learning and memory in AD mice	[95]
Natural	Review of the various animal models	A systematic review indicated that resveratrol supplementation improved cognitive function and reduced neuroinflammation in AD models. The benefits were attributed to the antioxidant properties and modulation of signaling pathways	[107]
Synthetic	APPswePS1dE9 transgenic mice	Trans-ε-viniferin, a resveratrol dimer, decreased amyloid deposits more effectively than resveratrol. Treatment also partially improved spatial memory decline, highlighting its therapeutic potential	[108]
Natural	Tg6799 mice	Resveratrol administration (60 mg/kg daily for 60 days) reduced amyloid plaque formation, decreased Aβ42 levels, and improved spatial memory performance	[109]
Natural	3xTg-AD mice	Dietary resveratrol (100 mg/kg from 2 to 12 months of age) prevented memory loss, reduced Aβ and Tau pathologies, and enhanced proteostasis mechanisms	[110]
Synthetic	Human subjects with mild to moderate AD	High-dose resveratrol (up to 2 g orally daily) was safe and penetrated the blood–brain barrier, with observed reductions in cerebrospinal fluid Aβ40 levels	[96]

**Table 4:** A list of clinical trials of resveratrol for Alzheimer’s disease.

Clinical Trial	Interventions	Type of Study	Number of AD Patients	Clinical Phase Status	Related Publication
NCT01504854	Resveratrol	Double-blind, placebo-controlled trial	AD 120 patients	Phase 2 completed	[116]
NCT00743743	Longevinex brand resveratrol supplement	Randomized, double-blind, placebo-controlled clinical trial	AD 50 patients	Phase 3 withdrawn	NA
NCT00678431	Resveratrol with Glucose and Malate	Randomized, double-blind, placebo-controlled trial	AD 16 patients	Phase 3 completed	[117]
NCT02502253	Resveratrol, extract from grape seed	Randomized	Mild Cognitive Impairment (MCI) and Prediabetes	Phase 1 completed	NA
NCT06470061	Resveratrol, Quercetin, and Curcumin (RQC)	Randomized	AD and Retinal Amyloid-β	Not yet recruiting	NA

NA—Not available. The data were retrieved from https://clinicaltrials.gov/search?cond=Alzheimer%20Disease&term=Resveratrol (Accessed on 12 April 2025).

## References

[1] Prince M., Wimo A., Guerchet M., Ali G.C., Wu Y.T., Prina M. (2015). World Alzheimer Report 2015: The Global Impact of Dementia An AnAlysIs of Prevalence, Incidence, Cost and Trends. Ph.D. Thesis.

[2] Inouye K., Pedrazzani E.S., Pavarini S.C.I. (2010). Alzheimer’s disease influence on the perception of quality of life from the elderly people. Rev. Da Esc. Enferm. Da USP.

[3] Karran E., Mercken M., Strooper B.D. (2011). The amyloid cascade hypothesis for Alzheimer’s disease: An appraisal for the development of therapeutics. Nat. Rev. Drug Discov..

[4] Selkoe D.J. (2001). Alzheimer’s disease: Genes, proteins, and therapy. Physiol. Rev..

[5] Selkoe D.J., Hardy J. (2016). The amyloid hypothesis of Alzheimer’s disease at 25 years. EMBO Mol. Med..

[6] Selkoe D.J. (2001). Clearing the brain’s amyloid cobwebs. Neuron.

[7] Harrison S.L., Lang C., Whitehead C., Crotty M., Ratcliffe J., Wesselingh S., Inacio C. (2020). Trends in Prevalence of Dementia for People Accessing Aged Care Services in Australia. J. Gerontol. Ser. A.

[8] Matthews F.E., Stephan B.C.M., Robinson L., Jagger C., Barnes L.E., Arthur A., Brayne C. (2016). A two decade dementia incidence comparison from the Cognitive Function and Ageing Studies I and II. Nat. Commun..

[9] Zhang W., Xiao D., Mao Q., Xia H. (2023). Role of neuroinflammation in neurodegeneration development. Signal Transduct. Target. Ther..

[10] Carabotti M., Scirocco A., Maselli M.A., Severi C. (2015). The gut-brain axis: Interactions between enteric microbiota, central and enteric nervous systems. Ann. Gastroenterol. Q. Publ. Hell. Soc. Gastroenterol..

[11] Ashique S., Mohanto S., Ahmed M.G., Mishra N., Garg A., Chellappan D.K., Omara T., Iqbal S., Kahwa I. (2024). Gut-brain axis: A cutting-edge approach to target neurological disorders and potential synbiotic application. Heliyon.

[12] Zheng Y., Bonfili L., Wei T., Eleuteri A.M. (2023). Understanding the Gut–Brain Axis and Its Therapeutic Implications for Neurodegenerative Disorders. Nutrients.

[13] Bekris L.M., Yu C.E., Bird T.D., Tsuang D.W. (2010). Genetics of Alzheimer Disease. J. Geriatr. Psychiatry Neurol..

[14] Bagaria J., Bagyinszky E., An S.S.A. (2022). Genetics, Functions, and Clinical Impact of Presenilin-1 (PSEN1) Gene. Int. J. Mol. Sci..

[15] Zekanowski C., Styczyńska M., Pepłońska B., Gabryelewicz T., Religa D., Ilkowski J., Kijanowska-Haładyna B., Kotapka-Minc S., Mikkelsen S., Pfeffer A. (2003). Mutations in presenilin 1, presenilin 2 and amyloid precursor protein genes in patients with early-onset Alzheimer’s disease in Poland. Exp. Neurol..

[16] Pinals R.L., Tsai L.H. (2022). Building in vitro models of the brain to understand the role of APOE in Alzheimer’s disease. Life Sci. Alliance.

[17] Kanekiyo T., Xu H., Bu G. (2014). ApoE and Aβ in Alzheimer’s disease: Accidental encounters or partners?. Neuron.

[18] Pires M., Rego A.C. (2023). Apoe4 and Alzheimer’s Disease Pathogenesis—Mitochondrial Deregulation and Targeted Therapeutic Strategies. Int. J. Mol. Sci..

[19] Liu E., Zhang Y., Wang J.Z. (2024). Updates in Alzheimer’s disease: From basic research to diagnosis and therapies. Transl. Neurodegener..

[20] Tosto G., Reitz C. (2013). Genome-wide Association Studies in Alzheimer’s Disease: A Review. Curr. Neurol. Neurosci. Rep..

[21] Shen L., Jia J. (2016). An Overview of Genome-Wide Association Studies in Alzheimer’s Disease. Neurosci. Bull..

[22] Aisen P.S., Cummings J., Jack C.R., Morris J.C., Sperling R., Frölich L., Jones R.W., Dowsett S.A., Matthews B.R., Raskin J. (2017). On the path to 2025: Understanding the Alzheimer’s disease continuum. Alzheimers Res. Ther..

[23] Andrade-Guerrero J., Santiago-Balmaseda A., Jeronimo-Aguilar P., Vargas-Rodríguez I., Cadena-Suárez A.R., Sánchez-Garibay C., Pozo-Molina G., Méndez-Catalá C.F., Cardenas-Aguayo M.D., Diaz-Cintra S. (2023). Alzheimer’s Disease: An Updated Overview of Its Genetics. Int. J. Mol. Sci..

[24] Hampel H., Hardy J., Blennow K., Chen C., Perry G., Kim S.H., Villemagne V.L., Aisen P., Vendruscolo M., Iwatsubo T. (2021). The Amyloid-β Pathway in Alzheimer’s Disease. Mol. Psychiatry.

[25] de Oliveira J., Kucharska E., Garcez M.L., Rodrigues M.S., Quevedo J., Moreno-Gonzalez I., Budni J. (2021). Inflammatory Cascade in Alzheimer’s Disease Pathogenesis: A Review of Experimental Findings. Cells.

[26] Rawat P., Sehar U., Bisht J., Selman A., Culberson J., Reddy P.H. (2022). Phosphorylated Tau in Alzheimer’s Disease and Other Tauopathies. Int. J. Mol. Sci..

[27] D’alessandro M.C.B., Kanaan S., Geller M., Praticò D., Daher J.P.L. (2025). Mitochondrial Dysfunction in Alzheimer’s Disease. Ageing Res. Rev..

[28] Dhapola R., Sharma P., Kumari S., Bhatti J.S., HariKrishnaReddy D. (2024). Environmental Toxins and Alzheimer’s Disease: A Comprehensive Analysis of Pathogenic Mechanisms and Therapeutic Modulation. Mol. Neurobiol..

[29] Suresh S., Singh S.A., Rushendran R., Vellapandian C., Prajapati B. (2023). Alzheimer’s disease: The role of extrinsic factors in its development, an investigation of the environmental enigma. Front. Neurol..

[30] Nasb M., Tao W., Chen N. (2024). Alzheimer’s Disease Puzzle: Delving into Pathogenesis Hypotheses. Aging Dis..

[31] Puranik N., Song M. (2025). Therapeutic Role of Heterocyclic Compounds in Neurodegenerative Diseases: Insights from Alzheimer’s and Parkinson’s Diseases. Neurol. Int..

[32] Van Dyck C.H., Swanson C.J., Aisen P., Bateman R.J., Chen C., Gee M., Iwatsubo T. (2023). Lecanemab in Early Alzheimer’s Disease. N. Engl. J. Med..

[33] Mummery C.J., Börjesson-Hanson A., Blackburn D.J., Vijverberg E.G.B., De Deyn P.P., Ducharme S., Jonsson M., Schneider A., Rinne J.O., Ludolph A.C. (2023). Tau-targeting antisense oligonucleotide MAPTRx in mild Alzheimer’s disease: A phase 1b, randomized, placebo-controlled trial. Nat. Med..

[34] Robles A. (2009). Pharmacological Treatment of Alzheimer’s Disease: Is. it Progressing Adequately?. Open Neurol. J..

[35] Hensley K. (2010). Neuroinflammation in Alzheimer’s Disease: Mechanisms, Pathologic Consequences, and Potential for Therapeutic Manipulation. J. Alzheimers Dis..

[36] Hersh D.S., Wadajkar A.B., Roberts N.G., Perez J.P., Connolly N., Frenkel V., Winkles J.A., Woodworth G.F., Kim A.J. (2016). Evolving Drug Delivery Strategies to Overcome the Blood Brain Barrier. Curr. Pharm. Des..

[37] Li J., Zheng M., Shimoni O., Banks W.A., Bush A.I., Gamble J.R., Shi B. (2021). Development of Novel Therapeutics Targeting the Blood–Brain Barrier: From Barrier to Carrier. Adv. Sci..

[38] Shi M., Chu F., Zhu F., Zhu J. (2022). Impact of Anti-amyloid-β Monoclonal Antibodies on the Pathology and Clinical Profile of Alzheimer’s Disease: A Focus on Aducanumab and Lecanemab. Front. Aging Neurosci..

[39] Brockmann R., Nixon J., Love B.L., Yunusa I. (2023). Impacts of FDA approval and Medicare restriction on antiamyloid therapies for Alzheimer’s disease: Patient outcomes, healthcare costs, and drug development. Lancet Reg. Health-Am..

[40] Ramanan V.K., Armstrong M.J., Choudhury P., Coerver K.A., Hamilton R.H., Klein B.C., Wolk D.A., Wessels S.R., Jones L.K. (2023). Antiamyloid Monoclonal Antibody Therapy for Alzheimer Disease: Emerging Issues in Neurology. Neurology.

[41] Dyer O. (2024). Aduhelm: Biogen abandons Alzheimer’s drug after controversial approval left it unfunded by Medicare. BMJ Br. Med. J..

[42] Kim B.H., Kim S., Nam Y., Park Y.H., Shin S.M., Moon M. (2025). Second-generation anti-amyloid monoclonal antibodies for Alzheimer’s disease: Current landscape and future perspectives. Transl. Neurodegener..

[43] Pardridge W.M. (2009). Alzheimer’s disease drug development and the problem of the blood-brain barrier. Alzheimer’s Dement..

[44] Pardridge W.M. (2020). Blood-Brain Barrier and Delivery of Protein and Gene Therapeutics to Brain. Front. Aging Neurosci..

[45] Pardridge W.M. (2020). Treatment of Alzheimer’s Disease and Blood-Brain Barrier Drug Delivery. Pharmaceuticals.

[46] Chen Y., He Y., Han J., Wei W., Chen F. (2023). Blood-brain barrier dysfunction and Alzheimer’s disease: Associations, pathogenic mechanisms, and therapeutic potential. Front. Aging Neurosci..

[47] Daneman R., Prat A. (2015). The Blood–Brain Barrier. Cold Spring Harb. Perspect. Biol..

[48] Forloni G. (2020). Alzheimer’s disease: From basic science to precision medicine approach. BMJ Neurol. Open.

[49] Arafah A., Khatoon S., Rasool I., Khan A., Rather M.A., Abujabal K.A., Faqih Y.A.H., Rashid H., Rashid S.M., Bilal Ahmad S. (2023). The Future of Precision Medicine in the Cure of Alzheimer’s Disease. Biomedicines.

[50] Behl T., Kaur I., Sehgal A., Singh S., Albarrati A., Albratty M., Najmi A., Meraya A.M., Bungau S. (2022). The road to precision medicine: Eliminating the One Size Fits All approach in Alzheimer’s disease. Biomed. Pharmacother..

[51] Di Meco A., Vassar R. (2020). Early detection and personalized medicine: Future strategies against Alzheimer’s disease. Prog. Mol. Biol. Transl. Sci..

[52] Mohd Sairazi N.S., Sirajudeen K.N.S. (2020). Natural Products and Their Bioactive Compounds: Neuroprotective Potentials against Neurodegenerative Diseases. Evid. Based Complement. Altern. Med..

[53] Tavan M., Hanachi P., de la Luz Cádiz-Gurrea M., Segura Carretero A., Mirjalili M.H. (2023). Natural Phenolic Compounds with Neuroprotective Effects. Neurochem. Res..

[54] Teixeira J.P., de Castro A.A., Soares F.V., da Cunha E.F.F., Ramalho T.C. (2019). Future Therapeutic Perspectives into the Alzheimer’s Disease Targeting the Oxidative Stress Hypothesis. Molecules.

[55] Samanta S., Chakraborty S., Bagchi D. (2024). Pathogenesis of Neurodegenerative Diseases and the Protective Role of Natural Bioactive Components. J. Am. Nutr. Assoc..

[56] Lim D.W., Lee J.E., Lee C., Kim Y.T. (2024). Natural Products and Their Neuroprotective Effects in Degenerative Brain Diseases: A Comprehensive Review. Int. J. Mol. Sci..

[57] Dziedziński M., Kobus-Cisowska J., Stachowiak B. (2021). Pinus Species as Prospective Reserves of Bioactive Compounds with Potential Use in Functional Food—Current State of Knowledge. Plants.

[58] Salehi B., Mishra A.P., Nigam M., Sener B., Kilic M., Sharifi-Rad M., Fokou P.V.T., Martins N., Sharifi-Rad J. (2018). Resveratrol: A Double-Edged Sword in Health Benefits. Biomedicines.

[59] Gambini J., Inglés M., Olaso G., Lopez-Grueso R., Bonet-Costa V., Gimeno-Mallench L., Mas-Bargues C., Abdelaziz K.M., Gomez-Cabrera M.C., Vina J. (2015). Properties of Resveratrol: In Vitro and In Vivo Studies about Metabolism, Bioavailability, and Biological Effects in Animal Models and Humans. Oxid. Med. Cell Longev..

[60] Caruso F., Tanski J., Villegas-Estrada A., Rossi M. (2004). Structural basis for antioxidant activity of trans-resveratrol: Ab initio calculations and crystal and molecular structure. J. Agric. Food Chem..

[61] Gülçin I. (2010). Antioxidant properties of resveratrol: A structure–activity insight. Innov. Food Sci. Emerg. Technol..

[62] Nowacka A., Śniegocka M., Smuczyński W., Liss S., Ziółkowska E., Bożiłow D., Śniegocki M., Wiciński M. (2024). The Potential Application of Resveratrol and Its Derivatives in Central Nervous System Tumors. Int. J. Mol. Sci..

[63] Takizawa Y., Nakata R., Fukuhara K., Yamashita H., Kubodera H., Inoue H. (2015). The 4′-Hydroxyl Group of Resveratrol Is Functionally Important for Direct Activation of PPARα. PLoS ONE.

[64] Tain Y.-L., Lee W.-C., Wu K.L.H., Leu S., Chan J.Y.H. (2018). Resveratrol Prevents the Development of Hypertension Programmed by Maternal Plus Post-Weaning High-Fructose Consumption through Modulation of Oxidative Stress, Nutrient-Sensing Signals, and Gut Microbiota. Mol. Nutr. Food Res..

[65] Baur J.A., Sinclair D.A. (2006). Therapeutic potential of resveratrol: The in vivo evidence. Nat. Rev. Drug Discov..

[66] Kiselev K.V. (2011). Perspectives for production and application of resveratrol. Appl. Microbiol. Biotechnol..

[67] Ngo T.H., Lee Y.J., Choi H., Song K.S., Lee K.J., Nam J.W. (2024). Evaluating the anticancer potential of *Polygonum multiflorum* root-derived stilbenes against H2452 malignant pleural mesothelioma cells. Fitoterapia.

[68] Cha J., Yun J.H., Choi J.H., Lee J.H., Choi B.T., Shin H.K. (2024). Preclinical Evidence and Underlying Mechanisms of *Polygonum multiflorum* and Its Chemical Constituents Against Cognitive Impairments and Alzheimer’s Disease. J. Pharmacopunct..

[69] Hasan M.M., Cha M., Bajpai V.K., Baek K.H. (2013). Production of a major stilbene phytoalexin, resveratrol in peanut (*Arachis hypogaea*) and peanut products: A mini review. Rev. Environ. Sci. Biotechnol..

[70] Sales J.M., Resurreccion A.V.A. (2009). Maximising resveratrol and piceid contents in UV and ultrasound treated peanuts. Food Chem..

[71] Rimando A.M., Kalt W., Magee J.B., Dewey J., Ballington J.R. (2004). Resveratrol, pterostilbene, and piceatannol in *Vaccinium* berries. J. Agric. Food Chem..

[72] Yang Y., Sun Y., Gu T., Yan Y., Guo J., Zhang X., Pang H., Chen J. (2024). The Metabolic Characteristics and Bioavailability of Resveratrol Based on Metabolic Enzymes. Nutr. Rev..

[73] Resveratrol D., Liu J., Shi D., Morkovin E., Litvinov R., Koushner A., Babkov D. (2024). Resveratrol and Extra Virgin Olive Oil: Protective Agents Against Age-Related Disease. Nutrients.

[74] Alkhalifa A.E., Al-Ghraiybah N.F., Kaddoumi A. (2024). Extra-Virgin Olive Oil in Alzheimer’s Disease: A Comprehensive Review of Cellular, Animal, and Clinical Studies. Int. J. Mol. Sci..

[75] Bartra C., Yuan Y., Vuraić K., Valdés-Quiroz H., Garcia-Baucells P., Slevin M., Pastorello Y., Suñol C., Sanfeliu C. (2024). Resveratrol Activates Antioxidant Protective Mechanisms in Cellular Models of Alzheimer’s Disease Inflammation. Antioxidants.

[76] Quadros Gomes B.A., Bastos Silva J.P., Rodrigues Romeiro C.F., dos Santos S.M., Rodrigues C.A., Gonçalves P.R., Sakai J.T., Mendes P.F.S., Varela E.L.P., Monteiro M.C. (2018). Neuroprotective Mechanisms of Resveratrol in Alzheimer’s Disease: Role of SIRT1. Oxid. Med. Cell Longev..

[77] Tamaki N., Cristina Orihuela-Campos R., Inagaki Y., Fukui M., Nagata T., Ito H.O. (2014). Resveratrol improves oxidative stress and prevents the progression of periodontitis via the activation of the Sirt1/AMPK and the Nrf2/antioxidant defense pathways in a rat periodontitis model. Free Radic. Biol. Med..

[78] Rahman M.H., Akter R., Bhattacharya T., Abdel-Daim M.M., Alkahtani S., Arafah M.W., Al-Johani N.S., Alhoshani N.M., Alkeraishan N., Alhenaky A. (2020). Resveratrol and Neuroprotection: Impact and Its Therapeutic Potential in Alzheimer’s Disease. Front. Pharmacol..

[79] Boocock D.J., Faust G.E.S., Patel K.R., Schinas A.M., Brown V.A., Ducharme M.P., Booth T.D., Crowell J.A., Perloff M., Gescher A.J. (2007). Phase I dose escalation pharmacokinetic study in healthy volunteers of resveratrol, a potential cancer chemopreventive agent. Cancer Epidemiol. Biomark. Prev..

[80] Capiralla H., Vingtdeux V., Zhao H., Sankowski R., Al-Abed Y., Davies P., Marambaud P. (2012). Resveratrol mitigates lipopolysaccharide- and Aβ-mediated microglial inflammation by inhibiting the TLR4/NF-κB/STAT signaling cascade. J. Neurochem..

[81] Ma T., Tan M.S., Yu J.T., Tan L. (2014). Resveratrol as a Therapeutic Agent for Alzheimer’s Disease. BioMed Res. Int..

[82] Ge J.F., Qiao J.P., Qi C.C., Wang C.W., Zhou J.N. (2012). The binding of resveratrol to monomer and fibril amyloid beta. Neurochem. Int..

[83] Marambaud P., Zhao H., Davies P. (2005). Resveratrol promotes clearance of Alzheimer’s disease amyloid-beta peptides. J. Biol. Chem..

[84] Deng H., Mi M.T. (2016). Resveratrol Attenuates Aβ25-35 Caused Neurotoxicity by Inducing Autophagy Through the TyrRS-PARP1-SIRT1 Signaling Pathway. Neurochem. Res..

[85] Jia Y., Wang N., Liu X. (2017). Resveratrol and Amyloid-Beta: Mechanistic Insights. Nutrients.

[86] Shati A.A., Alfaifi M.Y. (2019). Trans-resveratrol Inhibits Tau Phosphorylation in the Brains of Control and Cadmium Chloride-Treated Rats by Activating PP2A and PI3K/Akt Induced-Inhibition of GSK3β. Neurochem. Res..

[87] Schweiger S., Matthes F., Posey K., Kickstein E., Weber S., Hettich M.M., Pfurtscheller S., Ehninger D., Schneider R., Krauß S. (2017). Resveratrol induces dephosphorylation of Tau by interfering with the MID1-PP2A complex. Sci. Rep..

[88] He X., Li Z., Rizak J.D., Wu S., Wang Z., He R., Su M., Qin D., Wang J., Hu X. (2017). Resveratrol Attenuates Formaldehyde Induced Hyperphosphorylation of Tau Protein and Cytotoxicity in N2a Cells. Front. Neurosci..

[89] Surya K., Manickam N., Jayachandran K.S., Kandasamy M., Anusuyadevi M. (2023). Resveratrol Mediated Regulation of Hippocampal Neuroregenerative Plasticity via SIRT1 Pathway in Synergy with Wnt Signaling: Neurotherapeutic Implications to Mitigate Memory Loss in Alzheimer’s Disease. J. Alzheimer’s Dis..

[90] Jin S., Guan X., Min D. (2023). Evidence of Clinical Efficacy and Pharmacological Mechanisms of Resveratrol in the Treatment of Alzheimer’s Disease. Curr. Alzheimer Res..

[91] Feng X., Liang N., Zhu D., Gao Q., Peng L., Dong H., Yue Q., Liu H., Bao L., Zhang J. (2013). Resveratrol inhibits β-amyloid-induced neuronal apoptosis through regulation of SIRT1-ROCK1 signaling pathway. PLoS ONE.

[92] Pan L.F., Wang X.B., Xie S.S., Li S.Y., Kong L.Y. (2014). Multitarget-directed resveratrol derivatives: Anti-cholinesterases, anti-β-amyloid aggregation and monoamine oxidase inhibition properties against Alzheimer’s disease. Medchemcomm.

[93] Subramanian A., Tamilanban T., Kumarasamy V., Sekar M., Subramaniyan V., Wong L.S. (2024). Design, Synthesis, and Invitro Pharmacological Evaluation of Novel Resveratrol Surrogate Molecules against Alzheimer’s Disease. Chem. Biodivers..

[94] Martínez A. (2024). Synthesis, in vitro activity, and molecular docking of caffeic acid and resveratrol derivatives against Alzheimer’s disease-related enzymes. Med. Chem. Res..

[95] Wang Z., Gao C., Zhang L., Sui R. (2024). Novel combination of Olesoxime/Resveratrol-encapsulated exosomes to improve cognitive function by targeting amyloid β-induced Alzheimer’s disease: Investigation on in vitro and in vivo model. Inflammopharmacology.

[96] Turner R.S., Thomas R.G., Craft S., Van Dyck C.H., Mintzer J., Reynolds B.A., Brewer J.B., Rissman R.A., Raman R., Aisen P.S. (2015). A randomized, double-blind, placebo-controlled trial of resveratrol for Alzheimer disease. Neurology.

[97] Islam F., Nafady M.H., Islam M.R., Saha S., Rashid S., Akter A., Or-Rashid M.H., Akhtar M.F., Perveen A., Md Ashraf G. (2022). Resveratrol and neuroprotection: An insight into prospective therapeutic approaches against Alzheimer’s disease from bench to bedside. Mol. Neurobiol..

[98] Villaflores O.B., Chen Y.J., Chen C.P., Yeh J.M., Wu T.Y. (2012). Effects of curcumin and demethoxycurcumin on amyloid-β precursor and tau proteins through the internal ribosome entry sites: A potential therapeutic for Alzheimer’s disease. Taiwan. J. Obstet. Gynecol..

[99] Vingtdeux V., Giliberto L., Zhao H., Chandakkar P., Wu Q., Simon J.E., Janle E.M., Lobo J., Ferruzzi M.G., Davies P. (2010). AMP-activated protein kinase signaling activation by resveratrol modulates amyloid-β peptide metabolism. J. Biol. Chem..

[100] Zhang F., Wang Y.Y., Liu H., Lu Y.F., Wu Q., Liu J., Shi J.S. (2012). Resveratrol Produces Neurotrophic Effects on Cultured Dopaminergic Neurons through Prompting Astroglial BDNF and GDNF Release. Evid.-Based Complement. Altern. Med..

[101] Feng Y., Wang X.P., Yang S.G., Wang Y.J., Zhang X., Du X.T., Sun X.X., Zhao M., Huang L., Liu R.T. (2009). Resveratrol inhibits beta-amyloid oligomeric cytotoxicity but does not prevent oligomer formation. Neurotoxicology.

[102] Albani D., Polito L., Batelli S., De Mauro S., Fracasso C., Martelli G., Colombo L., Manzoni C., Salmona M., Caccia S. (2009). The SIRT1 activator resveratrol protects SK-N-BE cells from oxidative stress and against toxicity caused by α-synuclein or amyloid-β (1-42) peptide. J. Neurochem..

[103] Candelario-Jalil E., de Oliveira A.C.P., Gräf S., Bhatia H.S., Hüll M., Muñoz E., Fiebich B.L. (2007). Resveratrol potently reduces prostaglandin E2 production and free radical formation in lipopolysaccharide-activated primary rat microglia. J. Neuroinflammation.

[104] Rao Y.L., Ganaraja B., Suresh P.K., Joy T., Ullal S.D., Manjrekar P.A., Murlimanju B.V., Sharma B.G., Massand A., Agrawal A. (2024). Outcome of resveratrol and resveratrol with donepezil combination on the β-amyloid plaques and neurofibrillary tangles in Alzheimer’s disease. 3 Biotech.

[105] Agarwal T., Manandhar S., Harish Kumar B., Famurewa A.C., Gurram P.C., Suggala R.S., Sankhe R., Mudgal J., Pai K.S.R. (2024). Oxyresveratrol-β-cyclodextrin mitigates streptozotocin-induced Alzheimer’s model cognitive impairment, histone deacetylase activity in rats: In silico & in vivo studies. Sci. Rep..

[106] Shamsher E., Khan R.S., Davis B.M., Dine K., Luong V., Cordeiro M.F., Shindler K.S. (2024). Intranasal Resveratrol Nanoparticles Enhance Neuroprotection in a Model of Multiple Sclerosis. Int. J. Mol. Sci..

[107] Buglio D.S., Marton L.T., Laurindo L.F., Guiguer E.L., Araújo A.C., Buchaim R.L., Goulart R.A., Rubira C.J., Barbalho S.M. (2022). The Role of Resveratrol in Mild Cognitive Impairment and Alzheimer’s Disease: A Systematic Review. J. Med. Food.

[108] Freyssin A., Rioux Bilan A., Fauconneau B., Galineau L., Serrière S., Tauber C., Perrin F., Guillard J., Chalon S., Page G. (2022). Trans ε-Viniferin Decreases Amyloid Deposits With Greater Efficiency Than Resveratrol in an Alzheimer’s Mouse Model. Front. Neurosci..

[109] Chen Y., Shi G.W., Liang Z.M., Sheng S.Y., Shi Y.S., Peng L., Wang Y.P., Wang F., Zhang X.M. (2019). Resveratrol improves cognition and decreases amyloid plaque formation in Tg6799 mice. Mol. Med. Rep..

[110] Corpas R., Griñán-Ferré C., Rodríguez-Farré E., Pallàs M., Sanfeliu C. (2018). Resveratrol Induces Brain Resilience Against Alzheimer Neurodegeneration Through Proteostasis Enhancement. Mol. Neurobiol..

[111] Puranik N., Yadav D., Song M. (2023). Advancements in the Application of Nanomedicine in Alzheimer’s Disease: A Therapeutic Perspective. Int. J. Mol. Sci..

[112] Salla M., Karaki N., El Kaderi B., Ayoub A.J., Younes S., Abou Chahla M.N., Baksh S., El Khatib S. (2024). Enhancing the Bioavailability of Resveratrol: Combine It, Derivatize It, or Encapsulate It?. Pharmaceutics.

[113] Smoliga J.M., Blanchard O. (2014). Enhancing the Delivery of Resveratrol in Humans: If Low Bioavailability is the Problem, What is the Solution?. Molecules.

[114] Griñán-Ferré C., Bellver-Sanchis A., Izquierdo V., Corpas R., Roig-Soriano J., Chillón M., Andres-Lacueva C., Somogyvári M., Sőti C., Sanfeliu C. (2021). The pleiotropic neuroprotective effects of resveratrol in cognitive decline and Alzheimer’s disease pathology: From antioxidant to epigenetic therapy. Ageing Res. Rev..

[115] Tosatti J.A.G., Fontes A.F.D.S., Caramelli P., Gomes K.B. (2022). Effects of Resveratrol Supplementation on the Cognitive Function of Patients with Alzheimer’s Disease: A Systematic Review of Randomized Controlled Trials. Drugs Aging.

[116] Moussa C., Hebron M., Huang X., Ahn J., Rissman R.A., Aisen P.S., Turner R.S. (2017). Resveratrol regulates neuro-inflammation and induces adaptive immunity in Alzheimer’s disease. J. Neuroinflammation.

[117] Zhu C.W., Grossman H., Neugroschl J., Parker S., Burden A., Luo X., Sano M. (2018). A randomized, double-blind, placebo-controlled trial of resveratrol with glucose and malate (RGM) to slow the progression of Alzheimer’s disease: A pilot study. Alzheimer’s Dement. Transl. Res. Clin. Interv..

[118] Andrade S., Ramalho M.J., Pereira M.D.C., Loureiro J.A. (2018). Resveratrol brain delivery for neurological disorders prevention and treatment. Front. Pharmacol..

[119] Fonseca-Santos B., Chorilli M. (2020). The uses of resveratrol for neurological diseases treatment and insights for nanotechnology based-drug delivery systems. Int. J. Pharm..

[120] Ortega A., Chernicki B., Ou G., Parmar M.S. (2024). From Lab Bench to Hope: Emerging Gene Therapies in Clinical Trials for Alzheimer’s Disease. Mol. Neurobiol..

[121] Paul D., Agrawal R., Singh S. (2024). Alzheimer’s disease and clinical trials. J. Basic. Clin. Physiol. Pharmacol..

[122] Nasr M. (2016). Development of an optimized hyaluronic acid-based lipidic nanoemulsion co-encapsulating two polyphenols for nose to brain delivery. Drug Deliv..

